# Letter to the Editor: When ALT defines the study denominator—MASLD burden or metabolic liver-injury burden?

**DOI:** 10.1097/HC9.0000000000001051

**Published:** 2026-09-16

**Authors:** Xuehua Cheng, Mingyu Sun

**Affiliations:** 1 Shuguang Hospital Affiliated to Shanghai University of Traditional Chinese Medicine, Shanghai, China; 2 Department of Traditional Chinese Medicine Internal Medicine, Huadong Hospital, Fudan University, Shanghai, China; 3 Institute of Liver Diseases, Key Laboratory of Liver and Kidney Diseases, Institute of Liver Diseases, Shuguang Hospital Affiliated to Shanghai University of Traditional Chinese Medicine, Shanghai, China

To the editor,

We read with great interest the study by Ali et al,^1^ which draws attention to the rapidly increasing burden of metabolic dysfunction–associated steatotic liver disease (MASLD) and its complications within the Veterans Health Administration (VHA). The number of patients identified with MASLD increased from 78,082 in 2010 to 621,400 in 2021, accompanied by notable rises in cirrhosis and HCC. These findings provide an important warning about the growing impact of metabolic liver disease in routine clinical practice. At the same time, the interpretation of this increase depends substantially on how the MASLD population was defined.

1. *The denominator problem:* The main issue is the study denominator. In this study, MASLD was identified by at least 2 elevated ALT measurements together with at least 1 metabolic dysfunction trait. This is a practical definition of electronic health records, but it is not equivalent to identifying the full MASLD population. Repeated ALT elevation preferentially selects patients with biochemical liver injury, while patients with imaging-defined steatosis and normal aminotransferases may be missed. We therefore caution against interpreting the reported 504% increase in cirrhosis prevalence and 742% increase in HCC prevalence as the population-wide burden of MASLD complications. These estimates may more accurately reflect the burden within an ALT-enriched, metabolically at-risk population. Current MASLD criteria are based on hepatic steatosis and cardiometabolic risk, rather than aminotransferase elevation alone.^2^ AASLD guidance also emphasizes that normal aminotransferases do not exclude advanced NASH or clinically significant fibrosis.^3^ Where available, the authors could compare the current ALT-based definition with imaging-documented steatosis, fatty liver diagnostic codes, and metabolically at-risk patients with normal ALT. Concordant trends would support the robustness of the findings. Divergent trends would be equally informative—suggesting that the observed burden is concentrated in an “ALT-enriched” liver-injury phenotype rather than distributed across the broader MASLD population.
2. *Detection may be part of the trend:* The observed increase may also reflect changes in how actively liver disease was sought. Between 2010 and 2021, the use of FIB-4, abdominal imaging, AFP testing, HCC surveillance, and hepatology referral likely increased across the VHA. Absent adjustment for these changes in case finding, rising cirrhosis and HCC rates may be partly attributable to improved detection rather than disease accumulation alone. Annual trends in fibrosis assessment, liver imaging, AFP testing, and specialist referral would help place the complication curves in context.
3. *Prevalence is not progression:* The study describes how many patients with MASLD-related cirrhosis and HCC were identified each year. It does not directly show how often newly diagnosed MASLD progresses to these outcomes. A separate inception cohort could address that question more directly: among patients newly identified with MASLD, how many develop cirrhosis or HCC within 5 or 10 years? Cox and competing-risk models would be particularly relevant in an older veteran population, where death may occur before liver-related outcomes.

Despite these concerns, Ali and colleagues provide an important picture of the growing burden of MASLD within the VHA. What remains uncertain is whether this picture represents MASLD broadly or a more selected population identified through persistent ALT elevation. In our view, that distinction is central. Testing alternative case definitions and accounting for changing detection practices would make the study’s message both clearer and more clinically meaningful.
